# Protocol for the effectiveness evaluation of an antenatal, universally offered, and remotely delivered parenting programme ‘Baby Steps’ on maternal outcomes: a Born in Bradford’s Better Start (BiBBS) study

**DOI:** 10.1186/s12889-023-15111-1

**Published:** 2023-01-28

**Authors:** Kate E. Mooney, Tracey Bywater, Josie Dickerson, Gerry Richardson, Bo Hou, John Wright, Sarah Blower

**Affiliations:** 1 Department of Health Sciences, University of York, York, UK; 2 Bradford Institute for Health Research, Bradford, UK; 3 Centre for Health Economics, University of York, York, UK

**Keywords:** Antenatal, Parenting programme, Maternal mental health, Maternal-child sensitivity, Baby steps, Infant, Quasi-experimental, Routine data

## Abstract

**Background:**

Poor perinatal mental health and maternal sensitivity towards a child in the early years can carry a long-term cost to individuals and to society, and result in negative child outcomes such as poor mental health and social emotional issues. Despite the recognition of early intervention and prevention, there is mixed evidence regarding antenatal parenting interventions that aim to enhance perinatal mental health and maternal sensitivity to prevent negative child outcomes. ‘Baby Steps’ is a relationship-based antenatal and postnatal parenting programme. The service evaluated in this study is delivered in a low-income and ethnically diverse community via Better Start Bradford.

This study aims to assess whether the universally, and remotely delivered Baby Steps programme is effective in improving postnatal maternal sensitivity (primary outcome) and postnatal maternal mental health (secondary outcome) when compared to services as usual 6–10 weeks post-birth. It will also assess differences in birth outcomes, and differences in the prevalence of poor perinatal mental ill health through routine data. The feasibility of collecting cost and health related resource use data for a future economic evaluation will be explored.

**Methods:**

The study is a quasi-experimental evaluation in a single centre. All participants are drawn from Born in Bradford’s Better Start (BiBBS) interventional family cohort study. Intervention participants will be matched to a demographically comparable control group using propensity score matching. The required minimum sample is *n* = 130 (ratio 1:1) to detect a medium effect (± 2.35, *d* = .50) on the primary outcome—maternal-child sensitivity, using the Mothers Object Relations Scale Short Form (MORS-SF). Secondary outcomes include the Patient Health Questionnaire (PHQ-8), Generalised Anxiety Disorder assessment 7 (GAD-7), identification of poor perinatal mental health through routine data, and birth outcomes (delivery method, gestation period, low birth weight). Service delivery costs and health resource use will be gathered from routine data.

**Discussion:**

This study will evaluate the effectiveness of Baby Steps for enhancing maternal-child sensitivity and maternal mental health when delivered universally and remotely. The findings regarding programme effectiveness, process, and costs will be relevant for researchers, service commissioners, and service staff.

**Trial registration:**

This study was prospectively registered with ISRCTN (22/04/2022, ISRCTN12196131).

**Supplementary Information:**

The online version contains supplementary material available at 10.1186/s12889-023-15111-1.

## Background

The perinatal period refers to both pregnancy (the antenatal period) and the first 12 months after childbirth (the postnatal period) [37]. We use the terms ‘women’ and ‘maternal’ to refer to birthing parents, we recognise however that not all birthing parents are women. Perinatal mental health problems carry an estimated long-term cost to society of £6.6 billion for each 1-year cohort of births, with most of this cost relating to negative impacts on the child rather than the mother [4]. Perinatal mental health problems can have an adverse effect on maternal perceptions of infant temperament and mother–child bonding [18, 35], and on the cognitive, behavioural, and psychomotor development of their child [31, 36]. Improving perinatal mental health is therefore crucial for improving mother–child bonding, to foster the development of children’s attachment, social and emotional competence, cognitive functioning, physical health, and mental health [19, 36, 42].

Specifically within the postnatal period, postnatal depression affects an estimated 10–19% of women globally [29]. In the UK, 11% of women have a diagnoses or symptoms of postnatal depression, with increased risk for those living in the most deprived areas [55]. A systematic review revealed that women belonging to ethnic minority groups are at an increased risk of reduced identification and management of poor mental health in the perinatal period [41].

Parenting programmes are those which help parents and their children to develop positive behaviours and relationships [24]. Early parenting programmes that reduce parental psychological distress and improve parent–child relationships during the perinatal period have the potential to reduce the economic burden on society and improve outcomes for children [1, 2]. However, systematic reviews which have synthesised evidence about these interventions have shown mixed results depending on the outcome examined.

A systematic review and meta-analysis of maternal antenatal parenting education programmes found that programmes had a significant positive effect on maternal mental health outcomes of stress and self-efficacy, but not on maternal anxiety or depression. There was also no evidence that programmes improved maternal physical outcomes or foetal birth outcomes, but caesarean birth rates and epidural anaesthesia rates were lower [30]. Similarly, a systematic narrative review of psychological interventions delivered during the antenatal period found that the effectiveness of these interventions was mixed, highlighting the need for more research into effective interventions in the antenatal period to prepare parents for parenting [6].

A meta-analytic review of online parenting programmes found interventions demonstrated medium sized effects on both parent and child outcomes [38]. However, this review combined heterogeneous studies such as targeted and universal programmes, and cognitive, behavioural, and attitude outcomes. Further, this review was published prior to COVID-19. Since the COVID-19 pandemic, many services have had to adapt dynamically to both the changing guidelines and to service user’s willingness to engage with services.

Related to co-parenting, a meta-analysis of interventions that focus on promoting effective co-parenting during the transition to parenthood found that they had small positive effects on couple adjustment, parent stress, child and parent mental health, and reduced child abuse [40]. The most effective interventions started in pregnancy and continued through the perinatal period, with a minimum of 5 sessions. However, it remains unknown which groups of parents benefit most from what kind of intervention with regards to the level of their social risk (e.g. levels of deprivation, ethnic group).

### The Baby Steps programme

Baby Steps is a parenting programme that was developed by the UK’s National Society for Prevention of Cruelty to Children (NSPCC). Baby Steps was developed as a 9-session, relationship-based, antenatal and postnatal parent education programme designed for vulnerable and socially excluded parents who often face challenges and ‘overload’ in pregnancy and early parenting. In a pre-post study of Baby Steps, Coster et al., [17] found positive changes in measures of both maternal and paternal attachment and warmth, lower rates of adverse birth outcomes, and reductions in maternal and paternal anxiety. However, this study was not undertaken by independent researchers, and was not designed to ascertain causal effectiveness. A qualitative study found that both mothers and fathers felt more confident in their parenting and had developed stronger support networks and communication with their partners and babies [12]. While this preliminary evidence is encouraging, a robust and independent evaluation of Baby Steps is required to ascertain causal intervention effectiveness.

The current study evaluates the Baby Steps programme as delivered in the Better Start Bradford area by Action for Children. In response to the COVID-19 pandemic in March 2020, Baby Steps in Better Start Bradford moved to a remote delivery model with condensed programme content. For the condensed programme content, Baby Steps in Better Start Bradford have developed a logic model [15]. Baby Steps also moved from a targeted to a universal offer in February 2022, which removed all eligibility criteria from the programme to enable all parents in Better Start Bradford to attend (see method section for more details). Due to the local adaptation of Baby Steps, and the potential uptake of the remote, universal, model more widely, it is timely and crucial to establish the effectiveness of the model and explore its associated costs.

### Objectives

The current study will examine the causal effectiveness of the Baby Steps intervention in a real-world setting, within a socioeconomically deprived and ethnically diverse area in Bradford. Baby Steps is commissioned by Better Start Bradford and delivered by Action for Children. The authors of this study are independent of the delivery or design of the service.

This study will use a matched-control comparison group. The specific aims of this study are to (1) assess whether the universally and remotely delivered Baby Steps programme is effective in improving the primary outcome of postnatal maternal sensitivity and (2) the secondary outcome of postnatal maternal mental health when compared to services as usual at 6–10 weeks postnatal. This study also aims to (3) assess whether the effects of Baby Steps on postnatal maternal sensitivity are mediated by postnatal maternal mental health.

This study has additional objectives with outcomes nested in routinely linked data, however, it will be underpowered to ascertain causal effectiveness in these outcomes. We aim to (4) assess differences in birth outcomes (delivery type, weight, gestation, feeding) and (5) assess differences in the identification and prevalence of poor perinatal mental ill health inferred through routine data linkage across the two groups. Finally, the economic analysis of this study will (6) estimate the cost of the intervention (including delivery and training to deliver Baby Steps) and (7) assess the feasibility of collecting health related resource use data for a future full economic evaluation.

## Methods/Design

### Study design

The study is a quasi-experimental evaluation with an intervention and matched control group in a single centre. In the absence of guidelines for quasi-experimental protocols, we have used the Strengthening the Reporting of Observational Studies in Epidemiology (STROBE) [25] guidelines for reporting methods in this protocol (see Additional file 1: Appendix A).

### Study setting

This study is set in Bradford, a city in Northern England with high levels of socioeconomic deprivation and a large ethnic minority population [10]. Baby Steps is currently being delivered by Action for Children through the Better Start Bradford programme. Better Start Bradford is part of network of five ‘Better Start’ programmes around the UK funded by The National Lottery Community Fund with the aim to improve the life chances of over 60,000 children living in some of the poorest parts of England. Within Bradford, the initiative is delivered in three areas: Bowling and Barkerend, Bradford Moor and Little Horton (see https://www.betterstartbradford.org.uk/).

Born in Bradford’s Better Start (BiBBS) is an interventional family cohort study, which runs in parallel to Better Start Bradford and is designed to support effectiveness evaluations of Better Start Bradford’s early life interventions [21]. BiBBS is a part of the Born in Bradford (BiB) family of studies [53]. All mothers and children in the BiBBS cohort have in-depth baseline data captured during pregnancy, and consent to routine linkage to their health and education records as well as information about intervention participation. BiBBS includes an additional questionnaire to all mothers 6–10 weeks postnatally, which serves as the primary and key secondary outcomes for Baby Steps. As a part of the consent to BiBBS, mothers agree to the information about them and their child being used to evaluate interventions, either as intervention or control participants. A significant proportion of pregnant BiBBS women report mild to severe symptoms of depression (46%), and symptoms of anxiety (30%) [22].

### Intervention

Baby Steps is a ‘relationship-based’ antenatal parent education programme developed by the NSPCC (https://learning.nspcc.org.uk/services-children-families/baby-steps).

Baby Steps delivery begins around the 26th to the 30th week of pregnancy. The programme under evaluation differs to the original programme in that it is delivered to groups remotely but retains some face-to-face contact. To adapt to remote delivery, the service delivery team have condensed the intended nine individual sessions into six group sessions. There are two one-to-one initial sessions (one of which is in the participant's home, one of which is virtual) prior to the course commencing to help build relationships between families and practitioners. Then there are four online, remote, group sessions before the baby is born. After the baby is born there is another in person home visit, followed by two online group sessions. See Table 1 for an overview of the sessions and timings.

**Table 1:** Overview of programme content and timings

Content	Time of delivery
**Antenatal course components**	
Initial assessment and consent	Antenatal week 1; one-to-one home visit
Evaluation questionnaires	Antenatal week 2; one-to-one virtual visit
Relationships, respect and support	Antenatal weeks 3–6; 2 group sessions
Parent-infant interactions	Antenatal weeks 6–8; 2 group sessions
**Postnatal course components**	
Re-engage families and complete evaluation questionnaires	Postnatal week 1; one-to-one home visit
Healthy bodies, healthy minds (group sessions)	Postnatal week 2; 1 group session
Understanding babies’ development and practicalities of parenthood	Postnatal week 2; 1 group session

Baby Steps sessions are facilitated by an Early Years Practitioner and a health practitioner (midwife or health visitor). The curriculum and activities for each group follow a set pattern at specific stages of pregnancy and following each baby’s birth. As the programme is now being delivered universally, any pregnant person (< 28 weeks gestation) who is living in one of the three Better Start Bradford areas is eligible to be referred to Baby Steps.

### Participants

#### Recruitment

Pregnant women are invited to join BiBBS by trained researchers who are not involved in the woman’s clinical care. This recruit takes place predominantly at Bradford Royal Infirmary’s Glucose Tolerance Test (GTT) clinic at 24–28 weeks gestation, and secondary recruitment sources in the community. If they consent, they complete a baseline questionnaire and consent for linkage of routine health and education data.

Recruitment to Baby Steps occurs through midwives asking eligible mothers to provide consent for their contact details to be shared with the Baby Steps delivery team, who then make contact to arrange a home visit.

#### Eligibility

Study participants will be pregnant women who are also participating in BiBBS and enrolled on or after the 1^ s^ May 2022. Data will be collected at the level of the mother.

Eligibility for the intervention group are:

- Currently pregnant and have not exceeded 24 weeks gestation
- Lives in the Better Start Bradford area
- Have consented to a referral to Baby Steps

Eligibility for the propensity score matched comparison group are:

- Have a child aged between 0- and 3-months during service delivery of Baby Steps
- Not currently receiving, and have not already received, Baby Steps in the Better Start programme at any time
- Provided data in the BiBBS additional sweep at any time in the duration of service delivery

See statistical methods section for more information on how the matched control group will be created.

### Data collection procedures

The study will include all women recruited from 1^st^ May 2022, up until the end of the delivery of the service (anticipated to be at the end of 2023).

Pregnant women complete a detailed questionnaire when recruited to BiBBS regarding their prenatal attachment, mental health, socio-demographics, family circumstances and more. Data provided from this questionnaire will enable intervention and control groups to be matched, where individual intervention participants can be matched to > 1 control group participant(s) (see statistical methods section for details).

All BiBBS mothers will complete outcome measures at 6–10 weeks after birth. The data are collected either by phone or at a physical home visit. The questionnaires include: the Patient Health Questionnaire-8 (PHQ-8), Generalised Anxiety Disorder assessment-7 (GAD-7), and the Mothers Object Relations Scale-Short Form (MORS-SF) (see below for details).

### Outcomes

#### Primary outcome measure (maternal sensitivity)

##### Mothers Object Relation Scale—Short Form (MORS-SF) 

To measure maternal sensitivity, we use the Mothers Object Relations Scales (MORS). The MORS is a validated measurement tool of the relationship between a caregiver and their infant or child. It measures a caregiver’s perception of their infants/child’s thoughts, feelings, and intentions towards them. It assesses mothers’ models of their infants on two axes; the emotional warmth-coldness and the invasion-withdrawal of the infant towards the mother [39]. As the original scale has 44 items, we choose the ‘short-form’ of this measure with 14 items to reduce participant burden (MORS-SF). The MORS-SF has been found to have good psychometric properties in Hungarian and British samples, with associations to maternal depression and infant temperament [39].

#### Secondary outcomes measures (maternal mental health)

##### Patient Health Questionnaire (PHQ-8) and Generalised Anxiety Disorder Assessment (GAD-7) questionnaires

The PHQ-9 and GAD-7 are widely used tools to assess depression and anxiety, respectively [33, 47]. They have previously been used as outcome measures in parenting programme trials [8]. The PHQ-9 has been found to successfully differentiate between depressed and non-depressed mothers, supporting its use to screen for postpartum depression [26].

BiBBS uses the PHQ-8, which is also a widely used tool to assess depression and only differs to the PHQ-9 in that it omits Item 9 [34]. Item 9 of the Patient Health Questionnaire-9 (PHQ-9) queries about thoughts of death and self-harm. Validation studies have found that both the PHQ-9 and PHQ-8 have similar diagnostic properties [44, 54]. To compare the PHQ-8 outcomes, any participants with a PHQ-9 questionnaire will have data dropped for Item 9.

#### Birth outcomes

Data will be linked to routinely collected birth outcomes by maternity services and Health Visitors for both the intervention and the control group. We will look at descriptive differences across delivery method, gestation period, and baby weight. In the case of multiple births (e.g. twins) we will use only the first-born child’s data.

#### Proxy indicator for poor perinatal mental health

BiBBS has developed a proxy indicator for the identification of poor perinatal mental health which uses predetermined code lists to identify any indication of poor maternal mental health in maternity electronic healthcare records, health visitor and GP records throughout pregnancy and up to one year after birth. Indicators include positive perinatal mental health screening, or use of assessment tools such as PHQ-9/GAD-7, referrals to perinatal mental health services and relevant prescriptions. Coded data will be examined for indication of perinatal mental health, with reference to predetermined code lists. More information will be available in the statistical analysis plan.

#### Other outcomes

##### Economic outcomes

We will explore whether it is feasible to link the BiBBS dataset above with other datasets that contain information on health-related outcomes and resource use.

##### Process outcomes

The process evaluation will aim to include both mothers and co-parents. The service collects information on which sessions parents attend and the number of sessions they attend, and group level information about the number of sessions delivered per programme and the deliverer for each session. Parent satisfaction questionnaires are also collected at the end of the programme. This information will be used to further explore effectiveness findings.

A process evaluation will explore parent’s experience of the course, identify the outcomes they reported after attending, and identify the barriers and facilitators to engagement of parents. The plans for this study will be specified in a separate protocol.

### Statistical methods

In addition to information provided below a detailed statistical analysis plan is in development and will be preregistered on the Open Science Framework (OSF). All analyses will be run using Stata [49].

#### Propensity score matching

A matched control group to the intervention group will be created using the propensity score matching method. The propensity score is the probability of a subject to receive a treatment conditional on the set of covariates [7]. The propensity score method with ‘one-to*-many’* matching will be applied to maximise statistical power [3], as we can utilise data from the BiBBS cohort for mothers who do not participate in Baby Steps.

**Fig. 1:**
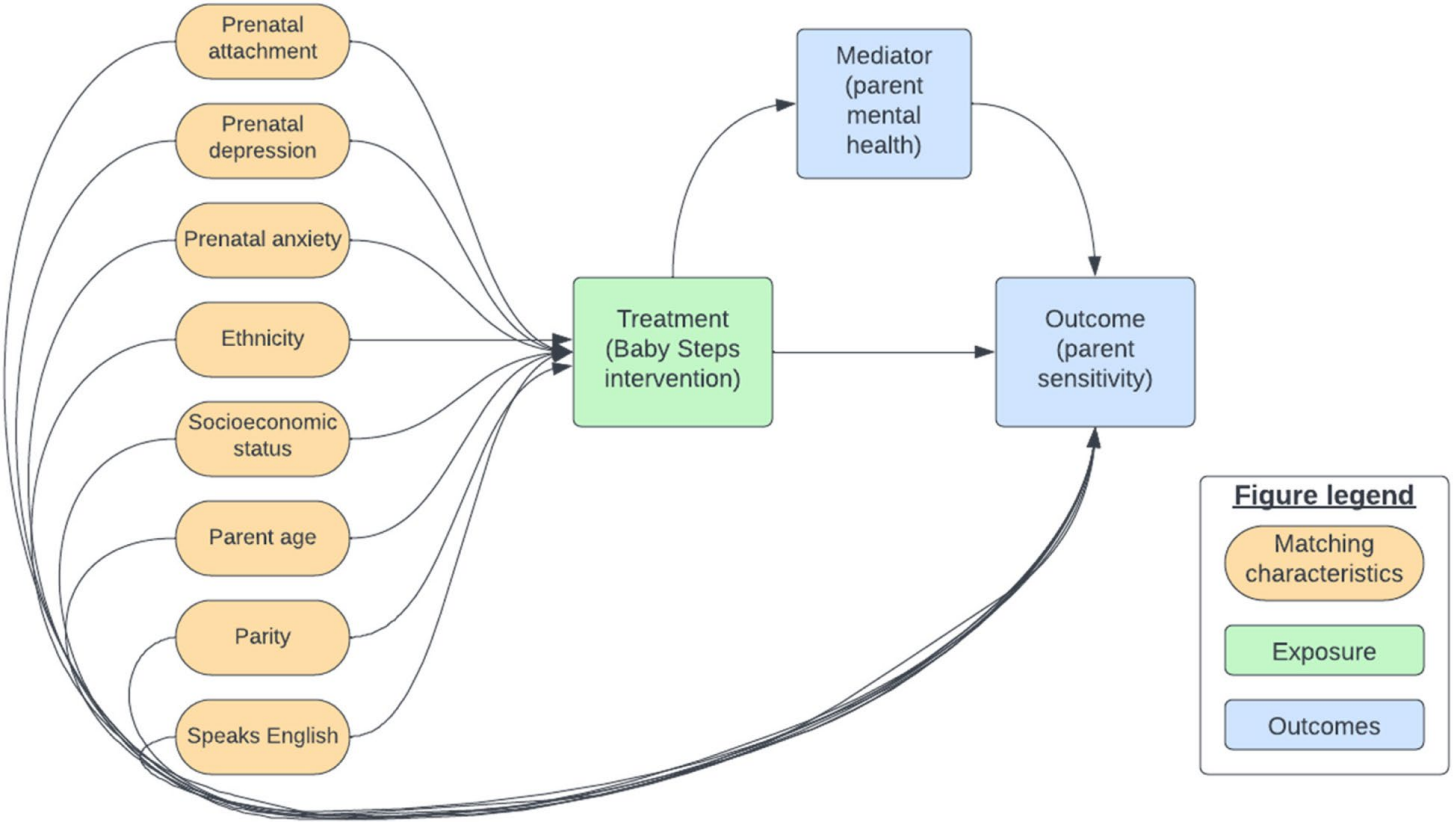
Directed Acyclic Graph of associations between confounders, treatment exposure, and primary outcome

Figure 1 presents a simplified Directed Acyclic Graph (DAG) created to identify confounding variables that are associated with both the treatment and the outcome [45, 52]. Figure 1 indicates the seven characteristics that we will match the groups on to estimate the effect of treatment on outcome.

Table 2 describes the covariates that we will use for propensity score matching obtained from the BiBBS baseline questionnaire completed before the child is born. All matching variables are at the level of the mother, not the child. Variables were selected to be matched upon if they were thought to have an association with both uptake of treatment, and with the outcomes (maternal sensitivity, maternal mental health), and there was empirical evidence to suggest this was the case (see references in Table 2).

**Table 2:** Covariates for matching obtained from BiBBS baseline questionnaire

Covariates (measurement tool if relevant)	Measurement	Justification
Prenatal attachment (Prenatal Attachment Inventory)	Continuous	[13]
Prenatal Depression (PHQ-8)	Continuous	[13]
Prenatal Anxiety (GAD-7)	Continuous	[13]
Ethnicity	Categorical	[13, 41, 51]
Socioeconomic position (financial security)	Continuous	[28, 48, 51]
Mother age	Continuous	[13, 28]
Parity (first child or not)	Continuous	[48]
Speaks English	Binary	[11, 51]

### Linear regression analyses 

After matched groups have been created, linear regression analyses will be run using group assignment as the independent variable, and the primary (maternal sensitivity) and secondary outcomes (maternal mental health) as the dependent variables. In addition to group assignment, two covariates will be included in the model: child age in days will be included as a continuous covariate, and Better Start Bradford area will be included as a categorical covariate with 3 levels. Models will be run for each outcome: maternal child sensitivity (MORS-SF), maternal depression (PHQ-8), and maternal anxiety (GAD-7).

We will conduct both Intention to Treat (ITT) analyses to ascertain the effects of enrolling in the intervention [23, 27], and per protocol analyses to ascertain the effects of receiving the treatment [50]. The ITT analyses will therefore include all mothers who enrolled in the intervention after an initial home visit, and the per protocol analyses will only include ‘completers’, defined as those who take part in at least 6 sessions (including at least one postnatal session).

### Mediation analyses

If the data have low levels of missingness, we will also conduct a mediation analysis to assess the hypothesis that the treatment effect on maternal sensitivity is partially mediated by maternal mental health. This analysis will use the propensity scores, as already described, and test the effect of this on maternal sensitivity via the maternal mental health outcomes *after* treatment.

### Economic analysis

The economic analysis will:

- Assess the feasibility of collecting health related resource use data through routine data and whether the quality of these data allow comparison of groups
- Assess the feasibility of collecting a range of consequences that could be used in a cost-consequence analysis
- Assess the feasibility of linking short term health outcomes measured in the datasets, to longer term costs and outcomes that are more appropriate for a full economic evaluation

Where routinely linked data allow, we will provide descriptive estimates of resource use and outcomes in each group. We will estimate the cost of the intervention and who that cost falls upon. We will explore the potential to extend the time horizon of the analysis by using an existing model to link short term study outcomes to lifetime as the benefits (and potentially costs) of the intervention are likely to impact far beyond the period assessed in the study.

#### Descriptive data

As birth outcomes and the proxy poor perinatal mental health outcomes are categorical, the required sample sizes for detecting differences in these outcomes is very large and unlikely to be obtained within this evaluation. We therefore do not expect to be able to assess effectiveness in these outcomes, but will report descriptive trends in birth outcomes, and in poor perinatal mental health for the intervention and control groups in our analysis.

#### Missing data methods

The most efficient method of handling missing data in propensity score matching depends on the mechanism of missing data in the matching variables, and whether there is unmeasured confounding. The missing indicator method and/or multiple imputation may be used depending on these factors for handling missing data in the matching variables [14]. If missing data are higher than 5% in any of the other variables (child age, maternal mental health and maternal sensitivity), then multiple imputation will be used to handle missing data [43]. If items are missing within any measurement tools, then missing data rules from the measure developer will be followed where available (e.g. within the PHQ-8). We will conduct a secondary analysis including only participants with complete data, and compare this to the primary analysis where missing data is handled. More detail will be provided on handling missing data in a statistical analysis plan.

### Sample size calculations

Sample size calculations for our primary and secondary outcomes were produced using Stata-17’s *power* command for the effectiveness outcomes. Sample size calculations were based on two sample means t-test with a two-tailed alpha of 5%.

#### Maternal-child sensitivity (MORS-SF)

In the absence of a minimum clinically important difference for the MORS-SF, we based our sample size calculations on 0.5 of a standard deviation in the MORS-SF [16]. A recent study of a trial delivered in Better Start Bradford indicates that the standard deviation in the MORS-SF is 4.7 [9]. We will have 80% power to detect 0.5 of this difference in the MORS-SF (± 2.35, *d* = 0.50) with 130 participants total (65:65), or if we obtain a larger control group through the BiBBS additional sweep (e.g. 140 total (56:84), 150 total (50:100), 180 total (45:135)). Historical data from Baby Steps in Bradford indicate that there is approximately a 40% attrition rate between enrolling and completing the project. To detect a difference in the MORS-SF, Baby Steps should therefore aim to enrol at least 72 participants to achieve the minimum sample size of 45 participants in the intervention group at the end of the study.

#### Maternal mental health (GAD-7 and PHQ-8)

Recent studies have estimated the minimum clinically important difference in the PHQ-9 to be 2 points [5, 32]. The sample size calculations show that we will have 80% power to detect this difference in the PHQ-8 and GAD-7 (± 2, *d* = 0.42) with 190 participants total (95:95), or if we obtain a larger control group through the BiBBS cohort (e.g. 200 total (80:120), 210 total (70:140), 250 total (62:187)). With the 40% attrition rate, Baby Steps should aim to enrol at least 99 participants to achieve the minimum sample size of 62 participants at the end of the study. We have therefore recommended to Baby Steps that they aim to recruit 99 intervention participants overall, as after accounting for attrition, this study should have statistical power to detect a minimum clinically important difference in all measures (MORS-SF, PHQ-9, GAD-7).

## Discussion

Early intervention and prevention can improve the developmental outcomes of children and reduce economic costs to society. However, there is mixed evidence for the effectiveness of antenatal parenting interventions to enhance maternal mental health and maternal-child sensitivity to prevent potential negative child outcomes.

Baby Steps is an antenatal and postnatal parenting programme which demonstrates promise [17], but requires robust evidence of effectiveness before it is adopted more widely. We considered the application of a Randomised Control Trial (RCT) design to evaluate the effectiveness of Baby Steps as a gold standard method for obtaining an unbiased estimate of effectiveness. However, the service delivery team had reservations about the implementation of an RCT, since this would involve substantial changes to their referral processes and service delivery methods. The implementation of an RCT could also have adverse effects on the sustainability of the service, as the referrals through other local services would have to be paused whilst the RCT was taking place. Instead, we apply a quasi-experimental design which is a pragmatic application to evaluate the effectiveness of interventions in real-world settings [46].

Since the COVID-19 pandemic, the delivery of programmes in an online format is growing. There is therefore a need to assess the effectiveness of these programmes and compare them to face-to-face delivery methods where possible. Whilst there is not yet any evidence for the effectiveness of the Baby Steps programme delivered using the in-person format, if the remotely delivered format is found to be effective, this may avoid the need for future in-person Baby Steps programmes. In-person programmes are likely to be more costly and time consuming for both delivery teams and participants. If the online format is found to be ineffective in this study, service delivery teams may need to consider returning to in person formats for parenting programmes—so that the in-person formats can also be evaluated.

A process and implementation evaluation is planned, and this will reveal more in depth information about how the service is implemented; which will be essential to understanding our findings [46]. We have planned to assess the feasibility of collecting information through routine data for an economic analysis, as it will not be possible in this study to collect additional economic measures to conduct a full economic evaluation. If Baby Steps is found to be causally effective in the outcomes we are collecting, a future study will need to conduct an economic evaluation to address whether the programme is cost effective.

The current study is nested within the BiBBS interventional family cohort study, which was primarily designed to examine the impact of individual, and stacked, interventions and services rather than conduct observational studies. Since 2016, BiBBS has worked in partnership with Better Start Bradford to engage the community and stakeholders, clarify the design of the interventions and monitor their implementation, harness routinely collected data, and develop toolkits and operational guides. The work from this partnership utilised innovative study designs and methods to assess process outcomes, clinical outcomes, and cost evaluations – all of which has informed design of the current study and other upcoming effectiveness studies [20]. This evaluation aims to test the causal effects of the Baby Steps programme on maternal mental health and maternal sensitivity. The study results will be submitted to a relevant journal and will be shared with the local service delivery team in Bradford, Baby Steps delivery teams more widely, and commissioners of these services.

## Supplementary Information

**Additional file 1.**

## Data Availability

These data cannot be shared publicly as they are available through a system of managed open access. Researchers are encouraged to make use of the BiBBS data, which are available through a system of managed open access. Before you contact us, please make sure you have read our Guidance for Collaborators. Our BiB Executive reviews proposals on a monthly basis and we will endeavour to respond to your request as soon as possible. You can find out about the different datasets in our Data Dictionary. If you are unsure if we have the data that you need please contact a member of the BiB team (borninbradford@bthft.nhs.uk). Once you have formulated your request please complete the ‘Expression of Interest’ form available here and send to borninbradford@bthft.nhs.uk. If your request is approved we will ask you to sign a Data Sharing Contract and a Data Sharing Agreement, and if your request involves biological samples we will ask you to complete a material transfer agreement.

## Abbreviations

BiBBS: Born in Bradford’s Better Start
PHQ-8: Patient Health Questionnaire – 8
GAD-7: Generalised Anxiety Disorder assessment 7
MORS-SF: Mothers Object Relations Scale – Short Form
ITT: Intention to Treat
DAG: Directed Acyclic Graph
RCT: Randomised Control Trial
